# An analysis of research grants allocated to researchers by the Smoking Research Foundation funded by Japan Tobacco Inc. in 2018

**DOI:** 10.18332/tpc/191140

**Published:** 2024-07-26

**Authors:** Rintaro Shiga, Shingo Nakagawasai, Eishin Hashimoto, Insu Cho, Hiroaki Saito, Akihiko Ozaki, Tetsuya Tanimoto, Shigeaki Ando, Hiroaki Shimmura, Michiya Saito, Takahiro Tabuchi

**Affiliations:** 1 Department of Radiology, Jyoban Hospital of Tokiwa Foundation, Fukushima, Japan; 2 School of Medicine, Peking University, Beijing, China; 3 Department of Internal Medicine, Soma Central Hospital, Fukushima, Japan; 4 Breast and Thyroid Center, Jyoban Hospital of Tokiwa Foundation, Fukushima, Japan; 5 Medical Governance Research Institute, Tokyo, Japan; 6 Department of Urology, Jyoban Hospital of Tokiwa Foundation, Fukushima, Japan; 7 Michiya Clinic, Fukushima, Japan; 8 Osaka International Cancer Institute, Osaka, Japan

**Keywords:** tobacco, Japan, smoking, investigation

**Dear Editor,**

The detrimental effects of tobacco use on health are a major concern, with an estimated 1.14 billion smokers worldwide as of 2019 and 7.69 million deaths annually^1^. One aspect of the tobacco industry that raises concerns is the possibility of covert marketing to the healthcare sector^2^, although the extent of this practice is not fully understood, particularly in Japan.

In 1986, the Smoking Research Foundation (SRF) was created when Japan Tobacco Inc. (JT) was established through the privatization of the Japan Tobacco and Salt Public Corporation. The research grants by the SRF may potentially play a role in preserving JT’s influence over the medical community through the distribution of research grants to medical professionals and researchers^3^. To shed light on this issue, we investigated the extent and distribution of the research grants paid by the SRF to medical professionals and researchers in 2018.

The data used for our analysis was sourced from the Annual Report of the SRF for fiscal year 2019, which was the only relevant document available to us. The Annual Report lists the names and affiliations of each project, which are categorized as either young or standard research projects.

The foundation provides funding up to 0.5 million Japanese Yen (JPY) (about 3111 sterling pound or GBP) annually for young researcher grants and up to 2 million JPY for general research projects. The exact monetary values of individual grants were not specified. Therefore, we assumed a full grant allocation of 0.5 million JPY for young researchers and 2 million JPY for general research for the sake of simplicity. When a grant covered multiple researchers, the amount was divided equally among all, including the primary investigators and other researchers.

We initially gathered all available data from the Annual Report, which included the names and affiliations of each project, along with the classification as either young or standard research. We then formatted this information into a database to facilitate further data collection on the Internet. Subsequently, the first and second authors conducted supplementary Internet searches to determine each researcher’s gender, specialty, qualifications, types of affiliations, and positions. We adhered to the classification of specialties as outlined in a previous article^4^. Regarding qualifications, individuals whose credentials did not clearly identify them as healthcare professionals based on their educational institutions or degrees were grouped together. In cases where any data were ambiguous, they consulted with the sixth author to clarify and finalize the details.

With regard to the analysis, we first calculated the total amount of research grants and average monetary value per recipient. Subsequently, we repeated this analysis for subgroups of each characteristic. The monetary value was converted from JPY into sterling pound (GBP) with an exchange rate of 160.7 JPY per 1 GBP. All the analyses were conducted using Microsoft Office Excel (Microsoft Corporation, Seattle, the United States).

Table 1 shows the distribution of research funding among the grant recipients. In total, 633 individuals received grants with a total amount of 2229148 GBP with a mean value of 3522 GBP (SD=2174.1). Of the recipients, 546 (86.3%) were male, and the most common characteristics were internal medicine (n=188; 29.7%) for medical specialty, medical doctor (n=332; 52.4%), and for qualification, national and public university (n=417; 65.9%) for affiliation, and professor (n=193; 30.5%) for position.

**Table 1:** Distributions of research funding among the grant recipients from the Smoking Research Foundation in 2018

*Characteristics*	*Number of people*		*Funding*		*Average (GBP)*	*SD*
	*n*	*%*	*GBP*	*%*		
**Total**	633	100	2229148	100	3521.6	2174.1
**Gender**						
Male	546	86.3	1945002	87.3	3562.3	2222.8
Female	81	12.8	268646	12.1	3316.6	1873.9
Unknown	6	0.9	15500	0.7	2583.3	231.1
**Specialty**						
Internal medicine	188	29.7	640398	28.7	3406.4	2107.5
Pharmacology	173	27.3	568230	25.5	3284.6	1625.8
Neurosynthesis	24	3.8	83576	3.7	3482.3	2604.4
Psychiatry psychosomatic medicine and psychology	20	3.2	62434	2.8	3121.7	2356.8
Obstetrics and gynecology	18	2.8	60884	2.7	3382.4	2407.9
Surgery	16	2.5	59272	2.7	3704.5	1811.2
Pathology	16	2.5	93620	4.2	5851.3	3377.2
Radiology	15	2.4	47120	2.1	3141.3	711.1
Physiology	11	1.7	35402	1.6	3218.4	1511.9
Nutritional science	10	1.6	31992	1.4	3199.2	1114.3
Oral surgery and dentistry	8	1.3	22072	1.0	2759.0	1418.2
Public health	8	1.3	46252	2.1	5781.5	3981.5
Anatomy	6	0.9	10850	0.5	1808.3	577.7
Head and neck surgery, and otorhinolaryngology	6	0.9	16120	0.7	2686.7	292.3
Neurosurgery	6	0.9	20336	0.9	3389.3	732.4
Biochemistry	5	0.8	15376	0.7	3075.2	1588.7
Pediatrics	5	0.8	24552	1.1	4910.4	3790.9
Anesthesia, emergency, and intensive care unit	4	0.6	9176	0.4	2294.0	569.9
Immunology	4	0.6	25172	1.1	6293.0	3764.6
Ophthalmology	3	0.5	7936	0.4	2645.3	643.0
Public health science	3	0.5	19592	0.9	6530.7	4170.0
Other	78	12.3	312108	14.0	4001.4	2324.3
Unknown	6	0.9	16678	0.7	2779.7	2433.2
**Qualification**						
Medical doctor	332	52.4	1150782	51.6	3466.2	2230.7
Healthcare professionals other than medical doctors	124	19.6	414718	18.6	3344.5	1750.1
Other than healthcare professionals	173	27.3	653728	29.3	3778.8	2330.4
Unknown	4	0.6	9920	0.4	2480.0	0.0
**Affiliations**						
National prefectural and other public universities	417	65.9	1494076	67.0	3582.9	2072.8
Private universities	139	22.0	486018	21.8	3496.5	2331.6
Research institutions	69	10.9	222580	10.0	3225.8	2499.8
Other	8	1.3	26474	1.2	3309.3	826.6
**Position**						
Professor	193	30.5	754416	33.8	3908.9	2366.5
Associate Professor	98	15.5	400458	18.0	4086.3	2757.1
Assistant Professor	93	14.7	286130	12.8	3076.7	1180.6
Director of the department	12	1.9	50034	2.2	4169.5	3750.4
Other	196	31.0	633082	28.4	3230.0	1907.9
Unknown	41	6.5	105028	4.7	2561.7	630.9

The study shows that in 2018, a research grant of more than 2 million GBP was allocated by the SRF, a JT-backed research organization. The funding was primarily allocated to health professionals and researchers who are considered authoritative, such as male medical doctors, national university professors, and those from prestigious institutions. These results, which have also been observed in previous studies on pharmaceutical industry payments in Japan^5^, are consistent with the hidden but original motivation behind establishing the SRF.

Given the substantial relationship between healthcare professional and researchers and the SRF, several proposals have been made to establish a system that enables researchers to conduct studies independently of such organizations. First, there is a need to secure sufficient private or public research funding. Secondly, it is necessary to educate healthcare professionals and researchers not to accept grants from tobacco-related organizations. Furthermore, grants related to smoking should be excluded from research grant introduction sites. Lastly, journals should not publish studies conducted with funding from sources associated with smoking.

Limitations of this survey include the fact that it is a single-year study and the specific monetary value of each grant is unknown. However, we believe that the methodology incorporated in this study is justifiable since the total monetary value of the research grant in 2021, which was reportedly 2.32 million JPY^6^, is similar to the speculated total amount of the grant in 2018. This indicates that a comparable amount of grant money was distributed from the SRF between 2018 and 2021.

## Data Availability

Data sharing is not applicable to this article as no new data were created.
